# Nanocellulose Grades with Different Morphologies and Surface Modification as Additives for Waterborne Epoxy Coatings

**DOI:** 10.3390/polym16081095

**Published:** 2024-04-14

**Authors:** Pieter Samyn, Patrick Cosemans

**Affiliations:** Department of Innovations in Circular Economy and Renewable Materials, SIRRIS, 3001 Leuven, Belgium; patrick.cosemans@sirris.be

**Keywords:** epoxy, nanocellulose, composites, coatings, structure, morphology, performance

## Abstract

While adding different micro- and nanocellulose types into epoxy coating formulations with waterborne phenalkamine crosslinker, effects on processing conditions and coating performance were systematically investigated. The variations in viscosity, thermal and thermomechanical properties, mechanical behavior, abrasive wear, water contact angles, and coating morphologies were evaluated. The selected additives include microcrystalline cellulose (MCC) at 1 to 10 wt.% and cellulose nanocrystals (CNC), cellulose nanofibers (CNF), cellulose microfibers (CMF), and hydrophobically modified cellulose microfibers (mCMF) at 0.1 to 1.5 wt.%. The viscosity profiles are determined by the inherent additive characteristics with strong shear thinning effects for epoxy/CNF, while the epoxy/mCMF provides lower viscosity and better matrix compatibility owing to the lubrication of encapsulated wax. The crosslinking of epoxy/CNF is favored and postponed for epoxy/(CNC, CMF, mCMF), as the stronger interactions between epoxy and CNF are confirmed by an increase in the glass transition temperature and reduction in the dampening factor. The mechanical properties indicate the highest hardness and impact strength for epoxy/CNF resulting in the lowest abrasion wear rates, but ductility enhances and wear rates mostly reduce for epoxy/mCMF together with hydrophobic protection. In addition, the mechanical reinforcement owing to the specific organization of a nanocellulose network at percolation threshold concentrations of 0.75 wt.% is confirmed by microscopic analysis: the latter results in a 2.6 °C (CNF) or 1.6 °C (CNC) increase in the glass transition temperature, 50% (CNF) or 20% (CNC) increase in the E modulus, 37% (CNF) or 32% (CNC) increase in hardness, and 58% (CNF) or 33% (CNC) lower abrasive wear compared to neat epoxy, while higher concentrations up to 1.5 wt.% mCMF can be added. This research significantly demonstrates that nanocellulose is directly compatible with a waterborne phenalkamine crosslinker and actively contributes to the crosslinking of waterborne epoxy coatings, changing the intrinsic glass transition temperatures and hardness properties, to which mechanical coating performance directly relates.

## 1. Introduction

Functional bio-based additives may replace common mineral- or fossil-based fillers in polymers, adhesives, and coatings, providing better performance, biodegradation, recyclability, or non-toxicity [1]. A first generation of fillers from natural plant fibers (e.g., bamboo, hemp, jute, palm, sisal, bagasse) was used in epoxy coatings for mechanical reinforcement [2]. Novel micro- to nanocellulose grades with various dimensions, shapes, and morphologies can be recovered from various sources [3,4,5] and replace traditional fillers such as graphite [6], graphene [7], carbon nanotubes [8], or silica [9]. Recently, the industrial production of nanocellulose was enabled in parallel with a reduction in mechanical energy needed for production [10] due to pre-processing [11] or the use of alternative solvents [12]. Many overview papers on nanocellulose materials are available [13], focusing on cellulose nanofibrils (CNF) [14], cellulose microfibrils (CMF) [15], or cellulose nanocrystals (CNC) [16]. They are produced in an aqueous suspension by mechanical fibrillation (e.g., grinding, milling, homogenization, microfluidization, ultrasonication) [17] and/or hydrolysis (e.g., chemical or enzymatic) [18]. Eventual surface modification can be considered to improve dispersibility [19], tune interface compatibility [20], or provide hydrophobic surface properties [21].

Epoxy resins are preferred binders for coatings, as their sustainability can be improved by using waterborne resins [22] or bio-based phenalkamine crosslinkers [23]. The reaction of an epoxide ring with primary amines or secondary amines results in the formation of a crosslinked polymer network [24], where curing kinetics are strongly altered in the presence of natural fillers [25]. The high degree of crosslinking determines a high chemical resistance, thermal stability, and better mechanical performance [26], while the glass transition temperature *T_g_* is used as a basic parameter that relates to the molecular structure [27] and mechanical properties [28]. The residual hydroxyl groups and ether bonds provide a high reactivity for adhesion with a substrate or interface reaction with additives. However, the performance of waterborne epoxy paints remains inferior compared to resin-based or solvent-based epoxy. In particular, they have a short pot life, low chemical resistance, limited mechanical strength, and poor corrosion protection. The film formation of a waterborne epoxy is a heterogeneous process regulated through the coalescence of water-dispersed particles [29], comparable to the drying mechanisms of other waterborne latex coatings [30]. Due to the hydrophilicity and high surface area of nanocelluloses, they are compatible with a waterborne epoxy and can interfere during crosslinking [31,32]: this likely results in homogeneous dispersion [33] and good mechanical properties [34]. In contrast, costly and tedious solvent-exchange processes are needed when traditional epoxy resins are used [35,36]. The interactions between nanocellulose and the epoxy matrix can introduce reversible hydrogen bridging or permanent crosslinking [37]. However, the high viscosity and aggregation cause problems in mixing nanocellulose at high concentrations [38]. These issues strongly depend on the morphology and percolation threshold of nanocellulose [39]. The surface modification is not prioritized to improve compatibility in waterborne systems, but it may reduce the viscosity and increase the dispersible volume fraction [40], while bringing additional functionality such as encapsulation and the controlled release of active ingredients [41], hydrophobicity [42], stabilization [43], or anti-microbial properties [44].

The reinforcement of epoxy composites with nano-, micro-, and mesoscale cellulose was highlighted in several studies, depending on the dispersibility and optimized concentrations [45]. In parallel, surface modification was recommended for better thermal, mechanical, and dynamic mechanical properties [40,46]. A concentration of 50 wt.% sulfated CNC was added to epoxy coatings to enhance corrosion protection [47], or 3 wt.% silanated CNC were added to improve hydrophobicity and corrosion protection [48]. The very low concentrations of 0.5 wt.% highly hydrophobic esterified CNC already provided better mechanical properties [49], in similar ranges of 0.2 to 5 wt.% concentrations of fatty-acid hydrophobized CNC [50]. The chemical modification also improved the micro-wear characteristics of CNC/epoxy nanocomposite coatings [51], while a general improvement in mechanical properties [52] and abrasive wear properties was reported for UV-crosslinked waterborne epoxy coatings with modified CNC [53]. In comparison, the low concentrations of up to 1 wt.% unmodified CNC also improved the mechanical strength [54], thermal behavior [55], and impact resistance [56], although proper dispersion was required and processing through solvent exchange was needed. The chemical, mechanical, physical, and thermal properties of the epoxy coatings with 0.2 to 0.7 wt.% pure CNC improved with very little aggregation [57], while further improvement with up to 2 wt.% CNC was observed after surface modification [58]. In parallel, the 1 to 2 wt.% pure CNC provided anti-corrosion protection to epoxy coatings [59,60]. After adding 3 wt.% pure CNF to epoxy coatings, better scratch resistance was observed [61]. Concentrations of up to 17 wt.% CNF could be added after surface modification to simultaneously increase mechanical, electrochemical, and tribological characteristics, but extremely long dispersions and sonication times were needed [62]. Also, the swelling of CNF during wetting with the epoxy matrix was limited after surface modification [63]. In particular, the influences of pure and modified CNF on curing kinetics and consequently the mechanical and tribological properties of bio-epoxy composites could be explained by structure–property relationships [64]: e.g., the mechanical and thermomechanical properties in waterborne epoxy improved for loadings of CNC up to 8 wt.% [65], CNC up to 15 wt.% [66], and CNF up to 1 wt.% [67]. In addition, better recyclability of epoxy/nanocellulose composites was proven after surface modification [68]. The above literature reveals that the morphologies and surface modification of different nanocellulose types highly influence the properties of epoxy composites.

Given the large variety in reported literature data depending on nanocellulose morphologies and concentrations and the lack of data for nanocellulose reinforcement in epoxy coatings (in contrast with epoxy bulk composites) with a waterborne phenalkamine crosslinker (in contrast with resin-based epoxy), this study provides a systematic investigation on the effects of different commercially available nanocellulose grades in the named system. In parallel, the compatibility with a unique type of surface-modified nanocellulose is investigated. The present paper demonstrates the performance of epoxy nanocomposite coatings including a range of appropriate nanocellulose concentrations, relating processing conditions (viscosity), intrinsic thermal and thermomechanical properties, mechanical resistance, and performance as a protective coating.

## 2. Materials and Methods

### 2.1. Materials

Different commercial micro- and nanocellulose grades with variable morphologies were added into an epoxy coating, including microcrystalline cellulose (MCC), cellulose nanocrystals (CNC), cellulose nanofibers (CNF), and cellulose microfibers (CMF), together with one grade of hydrophobically modified cellulose microfibers (mCMF), as prepared under lab-scale conditions according to the procedures in our previous work [69]. The MCC was used as a reference additive identified as an ultra-fine and highly pure cellulose powder with average mean particle size of 10 µm and size distribution between 8 and 20 µm (Arbocel UFC-100, JRS Rettenmaier & Söhne GmbH, Rosenberg, Germany). The CNC were received from Nanocrystacell (Stari trg pri Ložu, Slovenia) and added from a 4.6 wt.% aqueous stock suspension without prior dilution. The CNF grade Valida S191C was received from Sappi (Geleen, The Netherlands) and added from a 3 wt.% aqueous suspension. The CMF were received from VTT as a research-grade product from mechanically fibrillated softwood pulp and added from a 2.5 wt.% aqueous suspension. The mCMF were prepared as a 3 wt.% aqueous suspension by the simultaneous synthesis of hydrophobic nanocapsules from styrene–maleic anhydride in the presence of carnauba wax and CMF [69].

The waterborne epoxy coatings were formulated by reacting a stoichiometric ratio of DGEBA epoxy resin (EP101, Resion Resin Technology, Moordrecht, The Netherlands) with epoxy equivalent weight (EEW) = 200 g/mol, together with a waterborne phenal-kamine crosslinker (NX-8502, Cardolite, Ghent, Belgium) specifically developed with low viscosity (10,000–35,000 cps at 25 °C), a solids content of 44 wt.%, and a calculated active hydrogen equivalent AHEW = 424 g/mol. A theoretically calculated mass of 21.2 g phenalkamine crosslinker was added per batch of 10 g DGEBA resin.

### 2.2. Coating Formulation and Application

The epoxy/microcellulose coatings were prepared by adding different weight percentages of MCC (1, 2, 5, 7.5, and 10 wt.%), and the epoxy/nanocellulose coatings were prepared by adding different weight percentages of CNC, CNF, CMF, and mCMF (0.1, 0.2, 0.5, 0.75, 1, 1.5 wt.%) into the epoxy matrix through mixing in the aqueous suspension phase. Additive concentrations were calculated based on effective dry cellulose content. An overview of the samples is given in Table 1. The micro- or nanocellulose was first added as a dry powder (MCC) or from the mentioned aqueous suspensions (CNC, CNF, CMF, mCMF) into required weight fractions to the 21.2 g of waterborne phenalkamine crosslinker under high-speed (1000 rpm) mixing for 30 min (Dispermill Orange Series Laboratory Dispenser, ATP Engineering, Almere, The Netherlands), followed by sonication for 2 min (Ultra-Turrax T25, IKA Werke GmbH, Staufen, Germany), allowing for a full dispersion within the crosslinker. The crosslinker with additives was mixed with 10 g epoxy resin under mild stirring for a time of 5 min, as longer and/or more intense stirring introduced strong gelling.

The 10 × 10 × 5 cm^3^ softwood beech substrates (i.e., surface-planed panels from local DIY shop) were dried overnight at 60 °C in a hot-air circulating oven and coated with a 70 µm wet layer thickness by blade coating under a controlled speed of 5 mm/s, corresponding to a dry coating thickness of 68 ± 2 µm as controlled with a thickness gauge (Elcometer^®^ 456, Manchester, UK). The coatings were subsequently crosslinked for seven days at 25 °C and 60% relative humidity before further testing. The separate free-standing films with a thickness of 70 µm were cast in Petri dishes and simultaneously crosslinked under the same conditions as the coatings.

### 2.3. Characterization Techniques

The morphology of different nanocellulose grades was characterized by scanning electron microscopy, or SEM, (in the case of MCC) and atomic force microscopy, or AFM, (in the case of CNC, CNF, CMF, and mCMF). SEM was performed on a tabletop TM3000 (Hitachi, Krefeld, Germany), and AFM measurements were conducted in tapping mode on a Nanoscope III (Veeco, Santa Barbara, CA, USA) with a cantilever stiffness k = 50 N/m and a frequency of 300 kHz (PPP-NCH, Nanoandmore, Wetzlar, Germany).

The viscosity versus shear rate curves were measured according to ADTM D2196 using a DV-III Ultra viscosimeter with spindle SC4-27RD (Brookfield Engineering, Hadamar-Steinbach, Germany) at a controlled temperature of 25 °C and shear rates of 0.1 to 100 s^−1^. DSC, or differential scanning calorimetry, was performed on a DSC 3+ (Mettler Toledo, Zaventem, Belgium), inserting a liquid sample to follow the curing reaction as a function of temperature or a solid crosslinked sample to determine the glass transition temperature *T_g_*. The liquid samples of 4 mg were heated in hermetically sealed aluminum pans between 20 and 200 °C at 5 °C/min under a nitrogen atmosphere. The crosslinked coating samples of 7 mg were heated during two heating cycles between 20 and 110 °C at 10 °C/min under a nitrogen flow, while the thermal characteristics were determined from the second heating cycle. TGA, or thermogravimetric analysis, was conducted on a TGA-1 (Mettler Toledo, Columbus, OH, USA) during heating up to 650 °C at 50 °C/min, using a sample size of 5 mg under nitrogen gas with a flow rate of 60 mL/min. DMA, or dynamic mechanical analysis, was performed on an SDTA 861 (Mettler Toledo, Zaventem, Belgium) in uniaxial tension mode running a temperature sweep with a scanning range of 25 to 160 °C under 10 Hz frequency with a 3 °C/min heating rate, 0.1% strain, and 0.01 N preload. The storage modulus (E′) and loss modulus (E″) were recorded as a function of temperature, and the loss factor was calculated as tan δ = E″/E′. ATR-FTIR spectroscopy of the crosslinked coatings was conducted on a Nicolet iS 10 with diamond crystal (Thermo Fischer, Breda, The Netherlands).

A Taber tester with a circular rotary platform (Model 5130, Taber Industries, New York, NY, USA) and calibrated CS-10 abrasive wheels was used for abrasive wear testing according to ASTM D4060-10 [70], under a 250 g or 500 g load and 72 rpm rotational speed. After 1000 cycles, the weight loss was determined on an analytical balance with an accuracy of 0.001 g (Sartorius, Göttingen, Germany). A handheld Shore D micro-indenter with a standardized hardened steel tip of a 30° angle and 0.1 mm tip radius was used for hardness measurements according to ASTM D2240 [71]. A sclerometer type 3092 (Elcometer, Manchester, UK) with a tungsten carbide tip of 0.75 mm radius was used to measure scratch resistance according to ISO 4586-2 [72] under a load of 20 N. The scratches were optically evaluated with digital microscopy VHX-7000 (Keyence, Mechelen, Belgium) at low magnification, and the surface topography of worn coatings was visualized in more detail with a VK-X3000 laser interference microscope (Keyence, Mechelen, Belgium) at higher magnification. The OCA 50 goniometer (Dataphysics Instruments GmbH, Filderstadt, Germany) was used for static contact angle measurements with de-ionized water according to ISO 19403-2 [73], applying 3 µL droplets and describing their geometry with a tangent fit. The water contact angles were determined 10 s after the deposition of the droplet and averaged over 10 measurements per sample with an average standard deviation of ±2°. A ball impact tester was used to measure the absorbed energy according to ISO 6272 [74], reporting the impact strength (kJ/m^2^) on coatings as an average value from 10 repetitive tests with a standard deviation of 5%. A ball punch of defined weight and bottom geometry with a fixed diameter was dropped along a guiding rail from a given height while the deformed zone of the coating after impact was microscopically examined for cracks and flaking to determine the maximum impact energy.

## 3. Results and Discussion

### 3.1. Morphology of Micro- and Nanocellulose Grades

**Figure 1:**
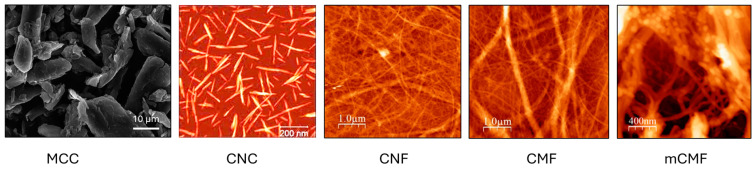
Morphology of different additives, including microcellulose grades characterized by SEM (MCC) and nanocellulose grades characterized by AFM (CNC, CNF, CMF, and mCMF).

The morphologies of the different nanocellulose grades used in this study were determined by SEM (MCC) or AFM (CNC, CNF, CMF, mCMF), as illustrated in Figure 1. The MCC can be referred to as microcellulose particles. The aspect ratio of the CNC grade is relatively high compared to other types of CNC, while the small fibrillar diameter of CNF indicates more intensive mechanical processing compared to CMF. The characteristics of mCMF with the deposition of styrene–maleimide nanocapsules with encapsulated carnauba wax onto the cellulose surface were fully described before [69]: the interactions between the nanoparticles and cellulose were attributed to hydrogen bonding between the residual anhydride groups of the nanoparticle shell and the hydroxyl groups of nanocellulose.

### 3.2. Viscosity Properties

**Figure 2:**
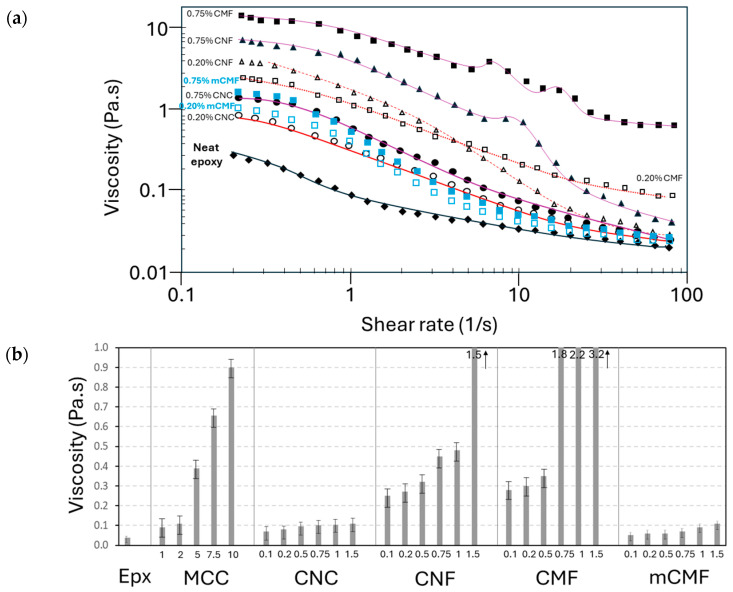
Viscosity measurements on liquid epoxy resin with micro- and nanocellulose additives: (**a**) shear viscosity curves as a function of shear rate for 0.2 and 0.75 wt.% concentrations and (**b**) representative viscosity values at a 5 s^−1^ shear rate for compositions with different additive types and concentrations (see numbers in wt.% on X-bar).

The processing and practical application conditions of epoxy resins with micro- or nanocellulose as a coating are strongly determined by the viscosity. The viscosity characteristics allow for the determination of conditions of good homogeneity and/or possible demixing of the additive under shear. The viscosity versus shear rate curves for epoxy resins with MCC, CNC, CNF, CMF, and mCMF at selected concentrations (0.2 and 0.75 wt.%) are shown in Figure 2a. In this study, a phenalkamine crosslinker with low viscosity was specifically selected because nanocellulose materials will generally augment the viscosity of the coating formulations. The viscosity increases over the entire range of shear rates in the presence of nanocellulose, but the differences between unfilled and filled epoxy are reduced at higher shear rates. The high viscosity under low shear rates can be problematic for processing, but a strong decrease in the viscosity at high shear rates for epoxy/CNF successfully demonstrates the intrinsic shear thinning properties of nanocellulose [75]. The gradual increase in the viscosity at higher nanocellulose concentrations indicates homogeneous mixing. Compared to other studies [76], a better dispersion of nanocellulose was presently experienced after premixing in the waterborne phenalkamine crosslinker. In contrast, the epoxy/nanocellulose nanocomposites were mostly formulated by direct mixing of the nanocellulose in the epoxy resin [77,78]. It is concluded that the viscosity of the epoxy resin is determined by the presence of additives, and the inherent properties of nanocelluloses with strong shear thinning become more pronounced as their concentration increases.

The representative viscosity values (e.g., 5 s^−1^ shear rate) of epoxy resin with different micro- and nanocellulose types and concentrations are compared in Figure 2b. The epoxy/CNC has the lowest viscosity due to possibilities for (self-)organization and alignment of short rod-like nanofibers under shear. When comparing the literature data on rheological features of pure CNC and CNF suspensions, it is confirmed that CNC have a lower viscosity than CNF in suspensions with the same concentration [75]. The epoxy/CNF offers a higher viscosity in parallel with strong shear thinning due to the more complex entanglements of CNF under shear. However, some transitions in the viscosity curves represent the partial agglomeration and disintegration of CNF at high concentrations. These transitions were also mentioned in the literature for viscosity curves of CNF suspensions and represent the Newtonian plateaus [79]. The latter fiber interactions become more pronounced for epoxy/CMF with high CMF concentrations, as the microfiber morphology is less homogeneous and more difficult to homogeneously disperse. However, the epoxy/CMF with low CMF concentrations has a lower viscosity under low shear possibly due to reduced interactions between CMF compared to CNF. The surface modification of mCMF significantly changes the viscosity profile of epoxy/mCMF, resulting in a lower viscosity over the full range of shear rates. The latter indicates reduced surface interactions and better compatibility of mCMF. The effect of mixing mCMF within an epoxy matrix for the formulation of nanocomposite coatings is demonstrated here for the first time. A comparative study of the rheological characteristics for pure CMF and mCMF suspensions was conducted before [80], indicating that the surface modification resulted in a lower viscosity of the suspensions and less variation in the viscosity depending on the degree of fibrillation. Otherwise, the incorporation of carnauba wax at the surface of mCMF also serves as a lubricant that reduces viscosity. The viscosities of epoxy/MCC have different ranges compared to nanocellulose additives and do not present shear thinning. In conclusion, the different morphologies of nanocellulose dominate the viscosity features of the epoxy/nanocellulose coating formulations, which strongly relate to the intrinsic properties of the nanocellulose suspensions. The nanocellulose additives are indeed known as rheological modifiers, resulting in the appropriate tuning of the viscosity of coating formulations depending on the application.

### 3.3. Thermal Analysis of the Epoxy Crosslinking

**Figure 3:**
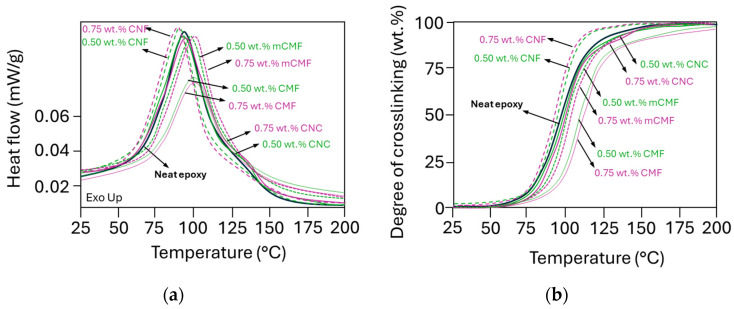
DSC measurements on liquid epoxy resin samples with different micro- and nanocellulose additives at 0.5 and 0.75 wt.% concentrations, including the (**a**) exothermal reaction peak and (**b**) degree of conversion.

The crosslinking and reaction kinetics of epoxy in the presence of phenalkamine and micro- or nanocellulose additives at selected concentrations (0.5 and 0.75 wt.%) were evaluated with DSC analysis. The exothermal reaction was monitored (Figure 3a), and the degree of crosslinking was calculated (Figure 3b).

The heterogeneity of epoxy nanocomposites mainly affects the initial stage of the crosslinking process [81]. Depending on the temperature shift of the maximum in the heat flow curve and the intensity of the exothermal reaction, the nanocelluloses can either catalyze the crosslinking reaction (i.e., lower maximum crosslinking temperature or higher peak intensity) or hinder the reaction (i.e., higher maximum crosslinking temperature or lower peak intensity). Depending on the nanocellulose grade, the addition of 0.5 and 0.75 wt.% CNF favors crosslinking, while CNC, CMF and mCMF either postpone the crosslinking or reduce the intensity of the exothermal peak and final degree of conversion. The enhanced crosslinking for epoxy/CNF reflects a better reactivity of CNF due to their large surface area and homogeneous dispersion within the epoxy matrix. The crosslinking of epoxy/CNC overlaps with the reaction of neat epoxy, as its surface area is smaller than the fibrillated nanocellulose owing to the intrinsic differences in the aspect ratio. The variations in reactivity also relate to the smaller effects on viscosity of epoxy/CNC compared to neat epoxy and epoxy/CNF, as demonstrated before. The reduced reactivity is most pronounced for epoxy/CMF, as the CMF with a lower degree of fibrillation may be less homogeneously mixed according to the viscosity profiles. The epoxy/mCMF presents the better compatibility of mCMF with the epoxy, and only slightly retards the crosslinking, as a lower amount of free hydroxyl groups is available at the surface. The latter are indeed partly occupied through the deposition of hydrophobic nanoparticles by physical interactions after surface modification [82]. Other studies have also confirmed that modified nanocellulose (e.g., after silanation) decreased the onset temperature of the reaction and accelerated the curing due to a lowering of the activation energy [64]. The evaluation of crosslinking by FTIR (see Electronic Supplementary Information, ESI, Figure S1) was complicated due to a strong overlap in the absorption bands related to different functional groups and low cellulose concentrations. Only slight variations in the 913 cm^−1^ band attributed to the ring-opening of the epoxide ring were noticed. Fundamental interactions between the phenalkamine crosslinker and epoxy were described before [83]. In conclusion, the fine morphologies of nanocellulose (CNF) promote the crosslinking of epoxy, while coarse nanocellulose grades (CMF) rather hinder crosslinking in parallel with a high viscosity, indicating that they are more difficult to mix. The surface modification (mCMF) obviously improves the compatibility for crosslinking but still confirms that access to free hydroxyl groups is crucial to catalyze crosslinking.

### 3.4. Thermal Transitions of Epoxy Nanocomposite Coatings

**Figure 4:**
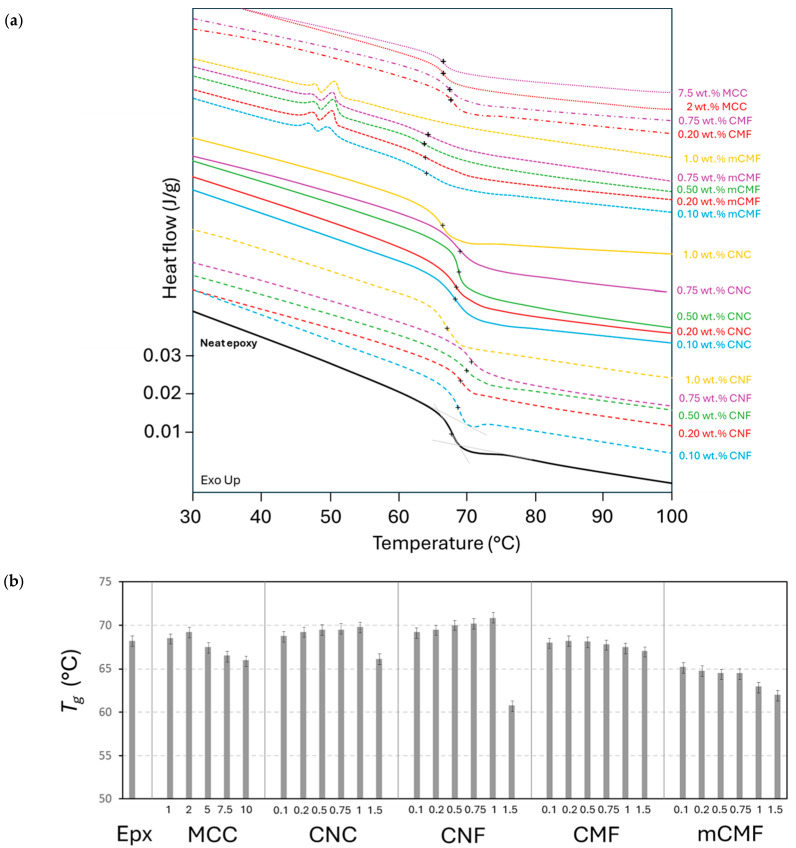
DSC measurements on cured epoxy coatings with different micro- and nanocellulose additives at various concentrations, including (**a**) heat flow curves with indication of *T_g_* position (+), (**b**) summary of *T_g_* values for additives at various concentrations (numbers in X-bar represent wt.%).

The variations in the *T_g_* for crosslinked epoxy in the presence of phenalkamine with micro- and nanocellulose additives were determined from the second temperature scan in DSC analysis (Figure 4a) and were summarized for different compositions (Figure 4b). The *T_g_* consistently varies depending on the type and concentrations of micro- or nanocellulose additives, indicating different interactions with the epoxy matrix. An increase in the *T_g_* indicates a suppressed motion of the polymer molecules in the case where an additive is promoting the crosslinking, while a decrease in the *T_g_* indicates enhanced mobility of the polymer molecules as the crosslinking is disturbed.

For epoxy/CNC and epoxy/CNF up to concentrations of 1 wt.%, the *T_g_* increases relatively to the native epoxy (*T_g_* = 68.2 °C), while the higher CNC and CNF concentrations become inhomogeneously mixed in the matrix and/or the high viscosity hinders the efficient diffusion of the reactive species for crosslinking. The results indicate that a less densely crosslinked polymer network at high concentrations of CNC or CNF improves molecular mobility and results in a reduction in the *T_g_*. Some studies indeed reported a plasticizing effect of randomly extracted nanocellulose particles added into the epoxy matrix [56], although this was previously also possibly ascribed to residual solvents. The *T_g_* of epoxy/CNF is higher than that of epoxy/CNC, indicating a more preferred morphology with a high aspect ratio of CNF resulting in a higher reactive surface area and accessibility of the reactive groups. For same reason, the progressively lower *T_g_* for epoxy/CMF might be a result of the lower crosslinking, mainly at the higher CMF concentrations.

For epoxy/mCMF, the more complex interactions between the mCMF and epoxy matrix lead to significant disturbance of the molecular crosslinking due to the occupation of reactive surface hydroxyl groups on the mCMF surface by hydrophobic moieties that cannot participate in the crosslinking reaction. In addition, the partial melting of the encapsulated wax is observed at 45 to 50 °C in the second DSC scan. Consequently, the carnauba wax has the role of a lubricant or plasticizer that enhances molecular mobility and reduces the *T_g_*. For epoxy/MCC, only a small increase in the *T_g_* is noticed up to 2 wt.%, as an indication of the lower reactivity of MCC compared to CNC or CNF nanocellulose, while the higher concentrations of MCC involve a high viscosity and disturbance of the crosslinking.

In conclusion, the higher *T_g_* for cured epoxy nanocomposite coatings is mostly promoted for epoxy/CNF, in line with the differences in reactivity depending on the additive morphologies. Although there is high variability in trends and most nanocellulose composites did not show a higher *T_g_* [84], a slightly increased *T_g_* for epoxy/CNC at low concentrations and decreased *T_g_* at higher concentrations was reported [78,85,86]. The increase in the *T_g_* for epoxy/CNC corresponds with previous studies indicating a temperature rise of 15 °C at 1.5 to 2.0 wt.% [59]. As explained in other literature, the decreases in the *T_g_* in epoxy nanocomposites can be explained through a reduction in the crosslinking degree [87], poor additive dispersion [88], or an additional free volume at the interface between the matrix and additives [89].

### 3.5. Thermal Stability of Epoxy Nanocomposite Coatings

**Figure 5:**
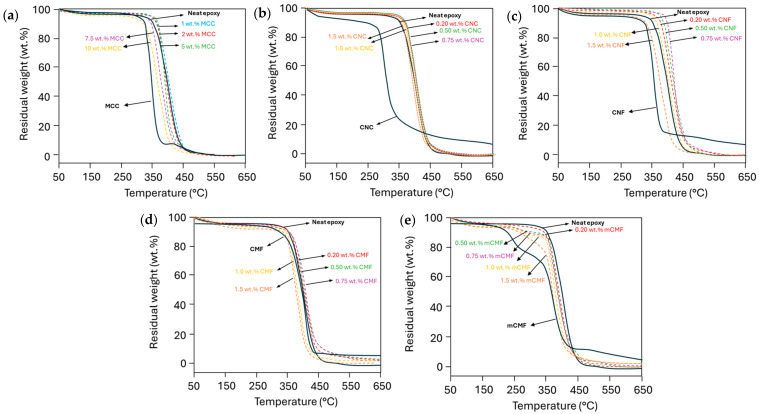
TGA measurements on cured epoxy coatings with different micro- and nanocellulose additives at various concentrations, including (**a**) MCC, (**b**) CNC, (**c**) CNF, (**d**) CMF, and (**e**) mCMF.

The thermal stability of epoxy nanocomposites with micro- and nanocellulose was measured by TGA and compared against neat epoxy (Figure 5). While the neat epoxy resin has higher thermal stability than the micro- and nanocellulose additives, the better thermal stability of epoxy nanocomposites depends on the nanocellulose type. In contrast, other studies reported lower thermal stability of epoxy filled with nanocellulose [40], owing to early degradation of unbound fragments or incomplete crosslinking.

The neat epoxy shows two degradation steps due to the degradation of the phenalamine (300 to 350 °C) and resin (400 to 450 °C). The cellulose materials present a small weight loss at temperatures of 100 to 130 °C, typically due to the evaporation of absorbed and intermolecular bonded water [90], while decomposition at higher temperatures is due to pyrolysis processes resulting in depolymerization, dehydration, decomposition of the glycosyl group, and char formation. The characteristic temperature ranges for each phase depend on the origin and type of nanocellulose, e.g., the initial pyrolysis ends at 338 °C for CNF and at 290 °C for CNC, while maximum weight loss occurs at 358 °C (CNF) or 320 °C (CNC). Thermal stability largely depends on surface characteristics and morphology, as eventual residual chemical groups on the CNC surface reduce thermal stability. As demonstrated before, the sulphated CNC act as an active component in an epoxy matrix [47], where the dehydration of cellulose molecules is catalyzed in the presence of sulphate groups and the decomposition of the inner crystal structure is affected. Alternatively, the higher thermal stability of CMF with an onset temperature of 348 °C and maximum weight loss temperature of 364 °C is in line with a lower degree of fibrillation. The lower surface area reduces the onset of thermal degradation, which typically initiates with the exposure of free surface groups. The weight reduction in mCMF is recognized as a multi-step thermal degradation corresponding to the presence of 25% carnauba wax degrading at 200 to 250 °C. In comparison, the thermal stability for MCC with an onset temperature of 336 °C and maximum weight loss temperature of 352 °C is due to the crystalline content in combination with a more compact structure and lower surface area compared to the nanocellulose materials.

For the epoxy nanocomposites, an increase in the thermal stability is noticed in the case of favorable interactions between nanocellulose and epoxy resulting in the formation of hydrogen bonds or better crystallization. The thermal stability for epoxy/CNF is superior for additive concentrations up to 0.75 wt.%, owing to the favorable interactions in parallel with the previously noticed increase in the *T_g_*. However, the lower thermal stability at 1.0 to 1.5 wt.% CNF has also been noticed before and attributed to the percolation threshold volume fraction causing increased thermal conductivity [55]. Alternatively, the drop in the thermal stability at high nanocellulose concentrations can be attributed to unreacted nanocellulose and epoxy after incomplete mixing. However, no separate degradation step of the nanocellulose is observed after homogeneous mixing in the epoxy matrix. The fine fibrillar morphology of CNF explicitly shows the best thermal stability, reflecting good interaction between the additives and epoxy matrix, except at high concentrations where the crosslinking might be reduced. The thermal stability for epoxy/CNC was almost not influenced by different CNC concentrations, as previous effects on the *T_g_* were also weaker compared to epoxy/CNF. In parallel, a decrease in the thermal stability for epoxy/CNC nanocomposites with an increasing CNC concentration was often reported in the literature [66]: here, the surface interactions were obstructed by the presence of intrinsic chemical groups (e.g., sulphates) on the CNC surface. For epoxy/mCMF, the presence of wax as a hydrophobic agent continued to influence the thermal stability at a low temperature, but this influence was not as large as that in the pure mCMF owing to the protective role of the wax and interfacial interactions between the modified cellulose fibrils and epoxy matrix. The thermal stability for epoxy/MCC can be explained in parallel with previous results indicating a lower *T_g_*, indicating that crosslinking reactions are hindered or retarded.

In conclusion, the thermal stability of epoxy resins depends on the morphology, surface characteristics, and concentrations of micro- and nanocellulose. A better thermal stability of the nanocomposite material generally indicates good interlocking, chemical bonding, and interactions between the additives and matrix. The latter requires additional thermal energy for degradation, as the interface region is generally a weak part where thermal degradation is locally initiated. In addition, the good dispersion of nanocellulose as a continuous three-dimensional network structure is favorable for better thermal stability, while mixing problems at higher concentrations lead to unbound nanocellulose and epoxy. In parallel with other literature on epoxy/nanocellulose foams, a homogeneous dispersion of nanocellulose improves the thermal stability and is enhanced for CNF [91]. Also, char formation enhances in the presence of nanocellulose, forming a protective layer with improved thermal stability above 450 to 500 °C [92].

### 3.6. Thermomechanical Properties of Epoxy Nanocomposite Coatings

**Figure 6:**
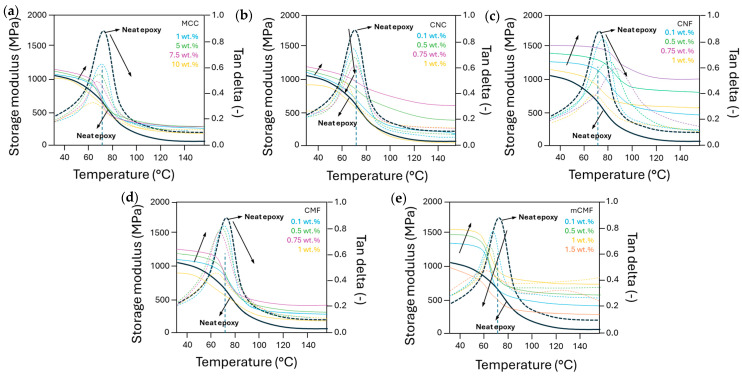
DMA measurements on cured epoxy resin samples with different micro- and nanocellulose additives at various concentrations, representing storage modulus E′ (full lines) and dampening factor tan δ = Ε″/Ε′ (dotted lines), including (**a**) MCC, (**b**) CNC, (**c**) CNF, (**d**) CMF, and (**e**) mCMF. Arrows indicate curve shifts as a function of concentration.

The DMA results of epoxy nanocomposites are presented in Figure 6 for compositions with MCC, CNC, CNF, CMF, and mCMF at different concentrations, including the storage modulus (E′) and dampening factor (tan δ = Ε″/Ε′) as a function of temperature. The transitions indicate regions of glassy and rubbery state with a transition point corresponding to the maximum in tan δ. The rise in the storage modulus E′ after the incorporation of given concentrations of micro- or nanocellulose additives indicates the successful reinforcement in mechanical properties. The concentration ranges with improved stiffness confirm the homogeneous dispersion and interaction of the additives within the epoxy matrix, resulting from a good interaction between the nanocellulose and epoxy matrix. The decrease in the mechanical properties at higher nanocellulose concentrations could be attributed to agglomeration or the formation of a too dense fibrillar network that hinders the crosslinking.

The storage modulus is highest for epoxy/CNF up to 0.75 wt.% and inferior for epoxy/CMF, while the effect of surface modification in epoxy/mCMF provides significant strengthening in the glassy range up to concentrations of 1 wt.%. The mechanical reinforcement of epoxy/mCMF in the rubbery region is superior to that of epoxy/CMF as an indication for sustained interactions at high temperatures. Especially at high temperatures where the strength of the polymer matrix is expected to weaken, a dense nanoscale fibrous network mostly dominates the mechanical properties. However, the mechanical modulus of epoxy/mCMF remains lower than that of epoxy/CNF likely due to the lubricating properties and possible release of encapsulated wax from mCMF. Different literature reports have mentioned that the reinforcing capability of CNC in epoxy is highly variable and strongly depends on the type of CNC and their inherent properties [78], but present results for epoxy/CNC are in comparable ranges of a 20 to 100 times improvement [66]. For epoxy/MCC, a lower storage modulus compared to nanocellulose reinforcements is noticed, as they lose strength at high temperatures and become comparable to neat epoxy. The lack of mechanical strengthening was also reported in previous studies, where epoxy macromolecules maintain a high mobility, as they are not in contact with the cellulose nanofibers and the interface cannot absorb shear forces unless fiber modification is performed [62]. In the present study, the reinforcement in the rubbery state is better compared to that in the previous reports of others [58], where a sudden loss of modulus and no strengthening was observed after the chemical treatment of the nanocelluloses. A parallel improvement in mechanical stiffness was noticed after hydrophobic modification of CNC by the grafting of fatty acids [50], together with an increase in toughness and elongation. The reasons for mechanical reinforcement through eventual post-crosslinking can be excluded, as the DSC measurements did not show additional exothermal events after crosslinking.

The dampening factor tan δ relates to secondary molecular relaxation mechanisms with significant shifts in peak temperatures depending on the nanocellulose types and concentrations. The increase in the relaxation temperature for epoxy/CNF and moderate increase for epoxy/CNC up to given concentrations is in line with the *T_g_* trends from DSC. The strong delay in relaxation for epoxy/CNF indicates that the distribution of a finely dispersed continuous fibrillar network efficiently enhances mechanical properties. Alternatively, the mechanical reinforcement and relaxation of epoxy/CNC relies more on the combination of interactions between both cellulose–cellulose and cellulose–epoxy at concentrations above the percolation threshold. The enhanced relaxation for epoxy/mCMF compared to epoxy/CMF may indeed refer to the formation of a smoothly lubricated interface between the cellulose fibrils and epoxy matrix in the presence of wax. The release of wax from mCMF has previously been noticed at temperatures of 130 to 150 °C [93] and is observed as a second tan δ maximum for epoxy/mCMF at 1.5 wt.%. The area below the tan δ peak represents the ratio of lost energy versus stored energy and is a measure for the mechanical dampening capacity of the material. The highest intensities are found for neat epoxy in parallel with high molecular mobility, while the reduced intensities in the presence of additives represent the uptake of mechanical loads by dampening. The high energy absorption of epoxy nanocomposites is a measure for the interface quality between epoxy and additives, which plays an important role in energy dissipation.

### 3.7. Mechanical Coating Properties

**Figure 7:**
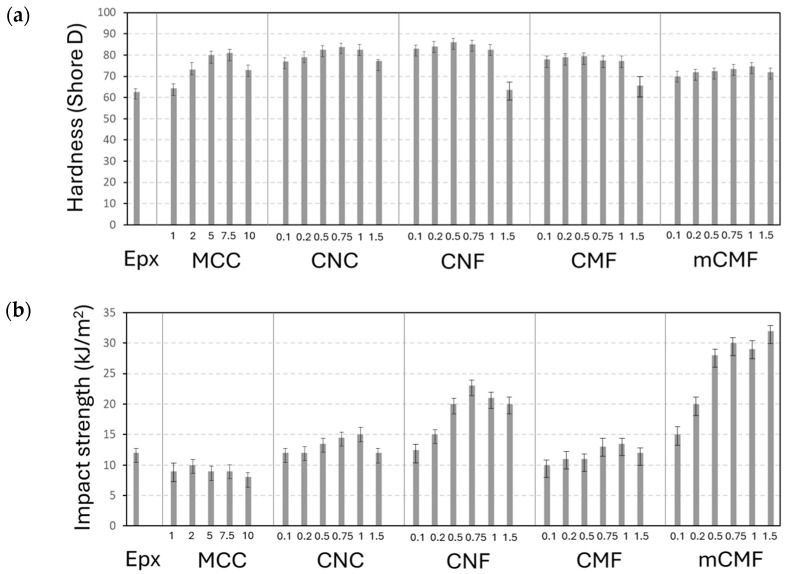
Mechanical testing results of epoxy coatings with different micro- and nanocellulose additives at various concentrations (numbers in X-bar represent wt.%), including (**a**) hardness and (**b**) impact strength.

The mechanical characteristics describing the performance of epoxy coatings were determined in relation to the inherent properties. The mechanical resistance is primarily related to the coatings’ hardness (Figure 7a) as a measure of resistance against plastic deformation and directly related to the degree of crosslinking [94]. The impact resistance (Figure 7b) can be related to ductility and energy absorption, in contrast with the occurrence of cracks after brittle fracture. It is known that neat epoxy coatings might easily crack and are prone to mechanical damage or wear [95], while the positive effects of nanocellulose additives on hardness and ductility are demonstrated below.

The hardness (Figure 7a) of almost all epoxy nanocomposite coatings is higher than that of neat epoxy coatings, while the variability in values remains relatively low. The latter represents a homogeneous dispersion of the additives and indicates that the higher hardness is related to a higher crosslinking density of the matrix rather than local mechanical reinforcement of nanoscale additives. However, the drop in hardness at the highest concentrations indicates the limitations for a uniform dispersion and possible retardance of the crosslinking, as also confirmed in other studies [96]. In other studies, a high variability in hardness in epoxy nanocomposites was attributed to a poor dispersion of nano-additives [97]. The highest improvement in hardness occurred for epoxy/CNF and was somewhat lower for epoxy/CNC. The latter agrees with variations in the *T_g_* according to DSC analysis, indicating the highest *T_g_* for coatings with the highest hardness. The intermediate hardness values for epoxy/CMF and lower hardness for epoxy/mCMF are also in line with the respective *T_g_* values. The explicit relationships between the hardness of epoxy coatings and the *T_g_* of the epoxy nanocomposites are shown in the Electronic Supplementary Information, Figure S2a.

The impact strength (Figure 7b) differs from hardness measurements, as loading under high speed introduces other fracture mechanisms and eventual debonding. The absorption of impact energy consists of a combination of plastic deformation of the matrix, fracture of the additives, or local debonding. While neat epoxy is relatively brittle [98], the nanoparticles are commonly used for toughening [99]. Previous studies demonstrated that micro- and nanoparticles enhance the impact strength through enhanced crosslinking while maintaining the continuous matrix phase at low concentrations [100], but disturbance in the matrix continuity deteriorates the impact strength at high concentrations [101,102]. For microparticle reinforcement in epoxy/MCC, the low impact strength and brittleness is generally explained by stress concentrations and micro-crack formation. The enhanced impact strength with nanocellulose additives signifies better energy absorption as it can be dissipated by several means through propagation within the fibrous structure. The toughening of epoxy/CNC relative to neat epoxy coatings has also been demonstrated in rigid adhesives [87] and in epoxy composites [56], where the critical stress intensity factor increased by 50% and 70% for 1.5 and 0.5% *w*/*w* cellulose acetate nanoparticles. As a high surface area is important in energy dissipation, a higher surface area of CNF is advantageous for the high impact strength. The epoxy/mCMF has a significantly higher impact strength, as ductility is favored by interfacial compatibility in presence of wax. Similar behavior was reported in previous studies [103], where interfacial toughening in flax/epoxy composites contributed to higher ductility and impact strength.

**Figure 8:**
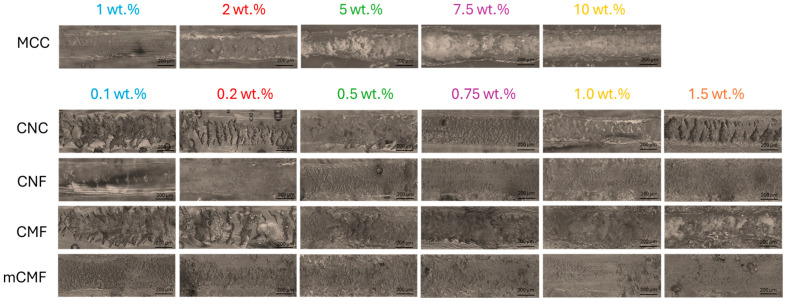
Scratching results of epoxy coatings with different micro- and nanocellulose additives at various concentrations, as observed through optical microscopy of the scratching track.

The scratch resistance (Figure 8) of epoxy coatings under a 20 N load is illustrated by microscopic images of the scratching track, with differentiation between ductile and brittle fracture morphologies. The brittle fracture for epoxy/MCC, epoxy/CNC, and epoxy/CMF is characterized by severe cracking and material displacement in front of the scratching tip. The ductile fracture for epoxy/CNF and epoxy/mCMF is characterized by plastic deformation resulting in smooth scratching tracks. The tendency for brittle and/or ductile fracture mechanisms relates to the previous impact strength and tan δ values as an indication of energy absorption. Moreover, the scratch resistance obviously improves in parallel with the high hardness of epoxy/CNF. It is indeed known that scratch resistance increases with crosslinking density and the higher *T_g_* of epoxy coatings [104], while pigments or wax can either increase or decrease the scratch resistance of an epoxy coating [105].

### 3.8. Wear Resistance and Protective Coating Properties

**Figure 9:**
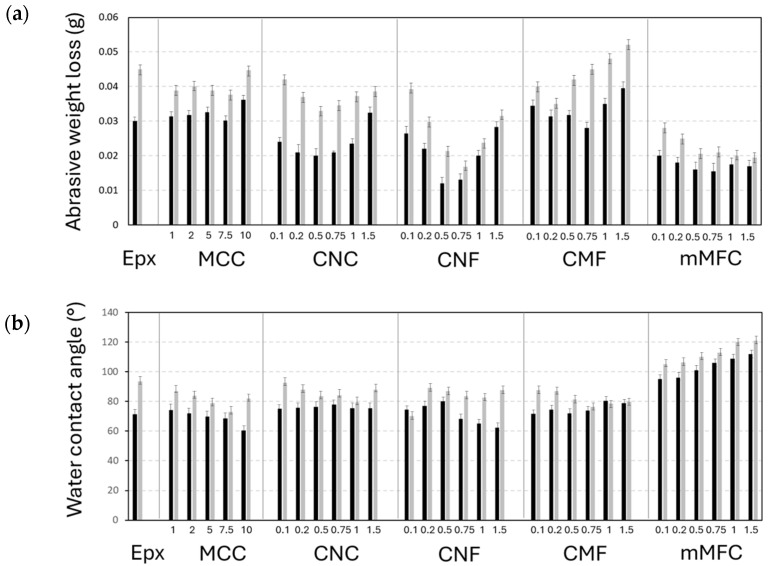
Protective properties of epoxy coatings with different micro- and nanocellulose additives at various concentrations: (**a**) abrasive wear measured as weight loss under low loads (black bar) and high loads (grey bar) and (**b**) static water contact angle on unworn coatings (black bar) and after wear under high loads (grey bar).

The abrasive wear resistance of epoxy coatings was evaluated with the total weight loss after testing under a low load (250 g) and a high load (500 g), as presented in Figure 9a. Depending on the morphologies and concentrations of micro- or nanocellulose, the wear resistance of the epoxy coating is not necessarily improved. For epoxy/CNF and epoxy/CNC (except at the highest concentrations), the abrasive wear is lower compared to neat epoxy in parallel with the hardness trends presented before: the high hardness for epoxy/CNF compared to epoxy/CNC is reflected in better abrasive wear resistance. Alternatively, the higher abrasive wear for epoxy/CMF compared to epoxy/CNC and epoxy/CNF is also in line with previous data indicating lower hardness. The explicit relationship between abrasive wear and hardness is shown in the Electronic Supplementary Information, Figure S2b. It is known that the hardness is a dominant parameter, as an increase in wear resistance with higher hardness was demonstrated for epoxy coatings [106]. Alternatively, the exceptionally low abrasive wear for epoxy/mCMF is influenced by the lubricating properties of the encapsulated carnauba wax. The present design of lubricating epoxy/mCMF coatings is comparable to epoxy coatings with incorporated oil-filled microcapsules, where the micro-encapsulation of linseed oil within a polyurethane shell showed self-lubricating properties and better tribological performance at microcapsule concentrations of 10 wt.% [107]. The surface morphologies of epoxy coatings after wear were further evaluated by optical and 3D topography images, as presented in Electronic Supplementary Information, Figure S3a,b.

The static water contact angles on epoxy coatings before and after wear are presented in Figure 9b. The neat epoxy coatings are hydrophilic and are expected to become more hydrophilic after adding cellulose. The hydrophilic properties are indeed enhanced for epoxy/MCC at high MCC concentrations, while water contact angles may rise in the presence of nanocellulose. The homogeneous dispersion of additives and embedding in the matrix prevent their exposure at the surface and the formation of a connected cellulose network for water penetration. Moreover, changes in the surface morphology and roughness (see below) through the exposure of nanofibrils at the surface could introduce slightly higher water contact angles. The irregular surface features on epoxy/CMF particularly caused higher water contact angles due to local pinning effects of the water droplet. The hydrophilic properties become prevalent for epoxy/CNF at higher additive concentrations and were not observed for epoxy/CNC: short CNC nanofibers can be better individualized compared to the long and more entangled CNF fibrils, not forming a continuously penetrating network for water. The water contact angles of epoxy/mCMF clearly increased after surface hydrophobization. While the latter effect was previously known for cast films of mCMF [62], their efficiency in hydrophobicity after incorporation within epoxy nanocomposites has not yet been demonstrated.

### 3.9. Morphology of Nanocomposite Coatings

**Figure 10:**
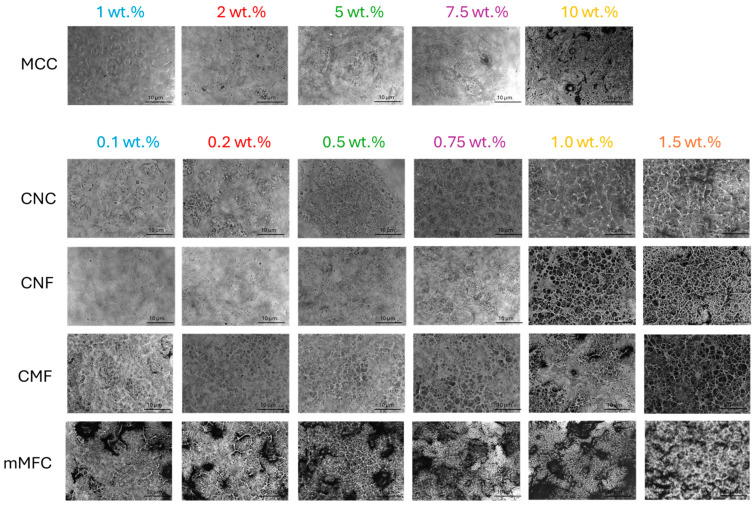
Detailed morphologies and arrangement of micro- and nanocellulose additives in unworn epoxy coatings.

**Figure 11:**
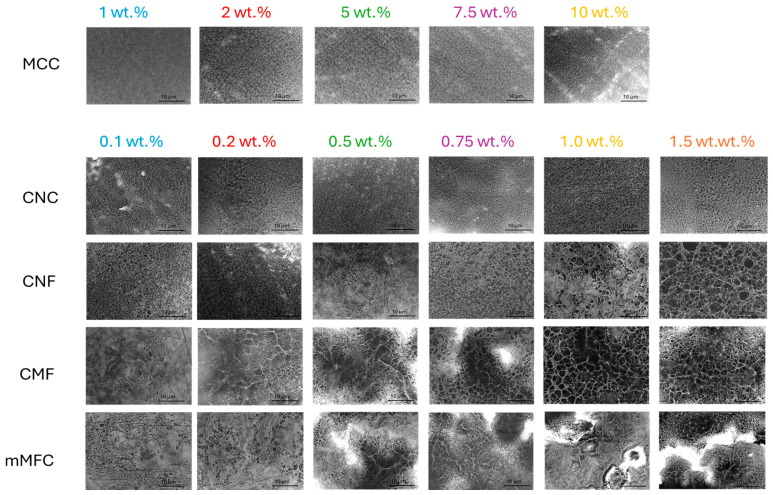
Detailed morphologies and arrangement of micro- and nanocellulose additives in worn epoxy coatings.

Microscopic analysis on the role of micro- and nanocellulose and their organization within the epoxy coating is shown in Figure 10 (coating before wear) and Figure 11 (coating after wear). With increasing additive concentrations, the surface aspects become more heterogeneous and dominated by the presence of fibers: at low concentrations, a homogeneous epoxy matrix phase is still observed, while this changes towards a more continuous nanofiber phase at high concentrations with eventually particular nanofiber organization and interactions.

For epoxy/MCC, the fibril aggregation above 10 wt.% may introduce deformation and internal stresses that reduce the mechanical properties of the coating. For epoxy/CNC, the fiber organization above 0.75 wt.% is observed with interactions between single CNC forming a continuous nanofiber network, while the size of the organized domains decreases at higher CNC concentrations. The interactions between cellulose nanofibers typically happen above a certain percolation threshold [108], while the self-organization of CNC into domain structures becomes evident [109]: it has been stated that from a practical standpoint, achieving and preserving this self-organization in a polymeric matrix represents an interesting route which is applicable to coat decorative materials. In parallel, an increase in dynamic moduli is observed above the percolation threshold for epoxy/CNC. The gradual exposure of CNC at higher concentrations indicates that the wettability with an epoxy matrix becomes more difficult, and regions with free nanofibers are created. The absence of an epoxy matrix is unfavorable for high hardness or low abrasive wear, and the critical concentration at 0.75 wt.% CNC corresponds to a limit for performance optimization. According to the literature, the matrix becomes less compact at high CNC concentrations, while microphase separation leads to more complicated mechanisms for energy dissipation [65]. For epoxy/CNF, a continuous nanofiber network forms above 1 wt.% with reduced wettability of the epoxy matrix, resulting in higher abrasive wear. For epoxy/CMF, the rough microfiber morphology presents a more open fiber network and agglomeration occurs above 1 wt.%, which results in higher abrasive wear and reduces mechanical properties. For epoxy/mCMF, the smooth surfaces indicate better dispersion and the formation of a densely entangled microfiber network. The presence of wax compensates for the lack of matrix wetting at high concentrations, thus avoiding the exposure of free nanofibers. On the worn surfaces, no separate fiber tear or pull-out was observed, indicating the protective action of embedded fibers with the enhanced resistance of the epoxy matrix. Although they depend on the size and aspect ratio of the nanocellulose, the results are comparable with previous concentrations ranges showing the homogeneous dispersion of nanocellulose for concentrations up to 2 wt.% without agglomeration [92]. This demonstrates good opportunities for uniform dispersion of hydrophilic nanocellulose within a waterborne phenalkamine. In conclusion, the good mechanical performance of the epoxy coatings corresponds with a homogeneous wetting of the additives by the matrix phase and possible self-organization above a threshold concentration.

## 4. Conclusions

This study presents new data for the formulation of epoxy nanocomposite coatings with micro- and nanocellulose additives, including a systematic screening of nanocellulose grades with different morphologies and hydrophobic surface modification. In particular, the compatibility of the additives mixed with a waterborne phenalkamine crosslinker allowed for the direct dispersion of micro- and nanocellulose in the aqueous crosslinker phase before mixing in a stoichiometric ratio with a DGEBA resin. It was demonstrated that the coating performance (i.e., abrasive wear) directly correlates to the intrinsic properties of the epoxy nanocomposite materials (i.e., glass transition temperature and hardness). As supported by thermal analysis, it is generally concluded that the nanocelluloses serve as reactive additives that interfere with the crosslinking process.

The processing properties are characterized by the increased viscosity of the epoxy nanocomposite coatings, but viscosity profiles are strongly influenced by the intrinsic properties of nanocellulose additives: i.e., (i) for CNF, shear thinning is most pronounced, and (ii) for mCMF, surface modification results in a lower viscosity over the full range of shear rates owing to the lubricating effect of the incorporated wax and reduced microfiber interactions. The reactivity of micro- and nanocellulose is illustrated by changes in the exothermal reaction during crosslinking, which is enhanced for CNF and postponed or reduced in intensity for CNC and CMF. The mCMF have improved compatibility with the epoxy and only slightly retard the crosslinking. The different interactions between additives and the epoxy matrix are illustrated by a *T_g_* shift, which increases in the presence of CNC and CNF up to given concentrations and decreases for the MCC, CMF, and mCMF additives. The different interactions between additives and the epoxy matrix are mainly proven by dynamic mechanical analysis, indicating that mechanical reinforcement is highest for CNF up to 0.75 wt.% and inferior for CMF, while the surface modification of mCMF provides significant strengthening in the glassy region up to 1 wt.%.

The mechanical properties of epoxy nanocomposite coatings indicated high hardness and impact strength for epoxy/CNF, offering a unique combination for improved toughness. The results for epoxy/CNF also provide better scratch resistance and lower abrasive wear rates. Through microscopic analysis, the formation of ordered nanocellulose structures and a nanofibrous network in the epoxy coating above the percolation threshold concentration limit corresponds to possible self-organization. A balance between the continuity of the nanofiber network and good wetting with the epoxy matrix is critical.

Based on this study, better selection of appropriate additive morphologies and a need for hydrophobic surface modification are illustrated, both changing the intrinsic properties of epoxy nanocomposites and the related coating performance.

## Figures and Tables

**Table 1:** Coating formulations of waterborne epoxy with micro- and nanocellulose additives.

Nanocellulose Type	Concentration (wt.%)	Resin Type
None	-	10 g DGEBA + 21.2 g NX-8102
MCC	1, 2, 5, 7.5, 10	
CNC CNF CMF mCMF	0.1, 0.2, 0.5, 0.75, 1, 1.5	

## Data Availability

Data are contained within the article.

## Supplementary Materials

The following supporting information can be downloaded at https://www.mdpi.com/article/10.3390/polym16081095/s1, Figure S1: Illustration of some FTIR spectra for neat epoxy, epoxy/CNF, epoxy/CNC, and epoxy/CMF with 0.75 wt.% nanocellulose concentrations; Figure S2: Relationships between intrinsic properties of composites of epoxy/micro- or epoxy/nanocellulose and coating performance: (a) relationship between hardness and glass transition temperature and (b) relationship between abrasive wear loss and hardness; Figure S3a: Surface morphologies (optical micrograph) and surface topography (3D scan) of worn epoxy coatings with different micro- and nanocellulose additives at various concentrations; Figure S3b: Detailed surface morphologies (optical micrograph) and surface topography (3D scan) of worn epoxy coatings with different micro- and nanocellulose additives at various concentrations.
