# Supplementary material for: Nanocellulose Grades with Different Morphologies and Surface Modification as Additives for Waterborne Epoxy Coatings
Source: Polymers (Basel). 2024 Apr 14;16(8):1095. doi: 10.3390/polym16081095 (PMC11054773; doi:10.3390/polym16081095)
Supplement: Supplementary file 1 [file polymers-16-01095-s001.zip › polymers-2957712-supplementary.pdf]

polymers

MDPI

Article

Nanocellulose Grades with Different Morphologies and Surface Modification as Additives for Waterborne Epoxy Coatings

Pieter Samyn * and Patrick Cosemans

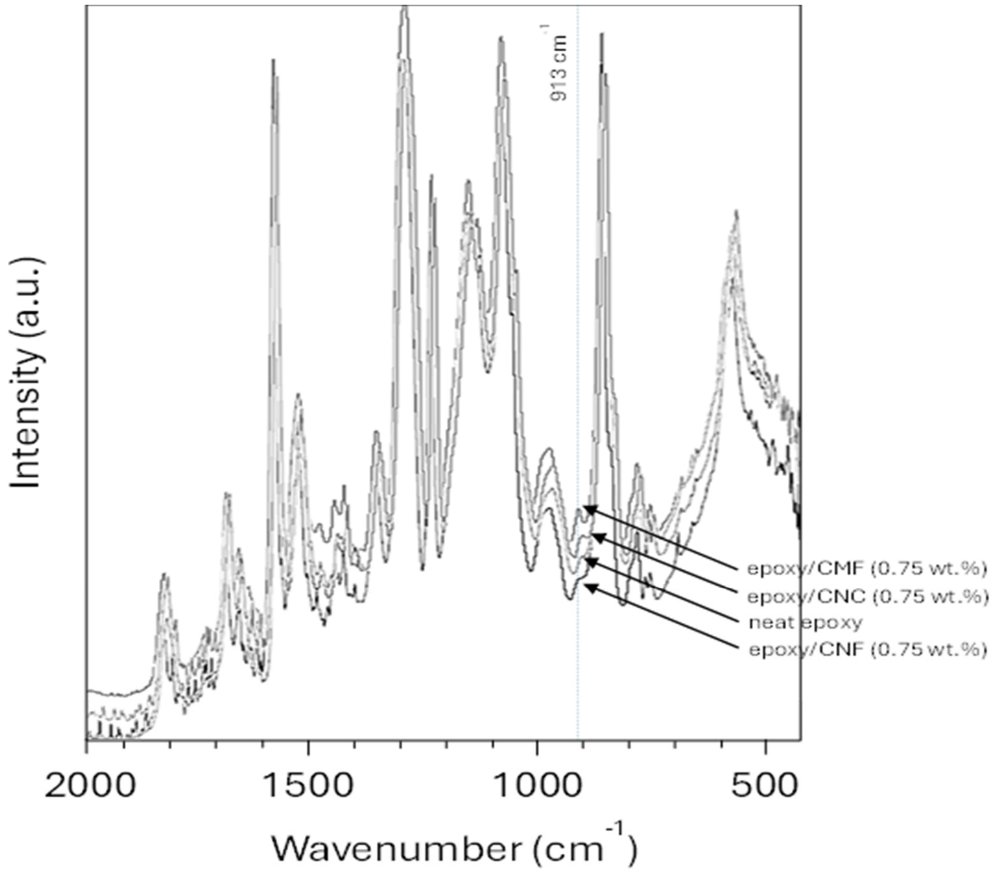

Figure S1. Illustration of some FTIR spectra for neat epoxy, epoxy/CNF, epoxy/CNC and epoxy/CMF with 0.75 wt.% nanocellulose concentrations.

(a)

(b)

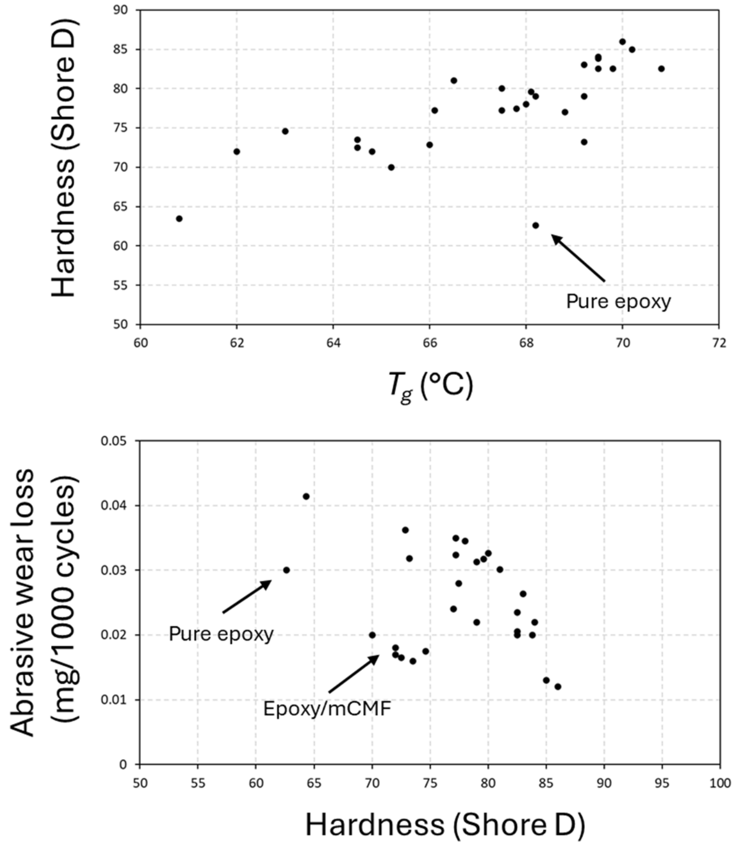

Figure S2. Relationships between intrinsic properties of composites of epoxy/micro- or epoxy/nano-cellulose and coating performance, (a) relationship between hardness and glass transition temperature, (b) relationship between abrasive wear loss and hardness.

As observed in Figure S3a and Figure S3b, the abrasive wear introduced different features depending on the type of cellulose additives, ranging from severe ductile abrasive wear towards more protective smooth sliding. For native epoxy coatings, the brittle abrasive features appear. For epoxy/MCC coatings, the rough surfaces and deep indentation of abrasive grooves are indeed a result of reduced hardness and ductile properties in parallel with previous scratching tests. The micro-ploughing wear and plastic deformation is a more severe form of abrasive wear for ductile materials, where material is displaced, and clear grooves are formed even under low loads. The deep abrasive grooves on worn on epoxy/MCC with 1, 2, 5 wt.% MCC correspond to high wear rates, while a more protective surface film formed at 7.5 wt.% MCC with consequently reduced wear. For nanocellulose additives, more ductile wear features are observed for epoxy/CNC compared to epoxy/CNF in parallel with the lower relative hardness for epoxy/CNC coatings. The most severe tear and ploughing wear are observed for epoxy/CNC coatings at 0.50 wt.% CNC, while a more protective surface film formed at 0.75 wt.% CNC. Overall, the higher hardness of epoxy/CNF compared to epoxy/CNC coatings results in only superficial surface scratches while large areas of the coating remain intact, especially at the higher concentrations of CNF. The lower hardness and severe wear of epoxy/CMF coatings is characterized by severe ploughing wear. The lubricating properties of epoxy/mCMF coatings present smooth wear surfaces with more tendency for plastic deformation.

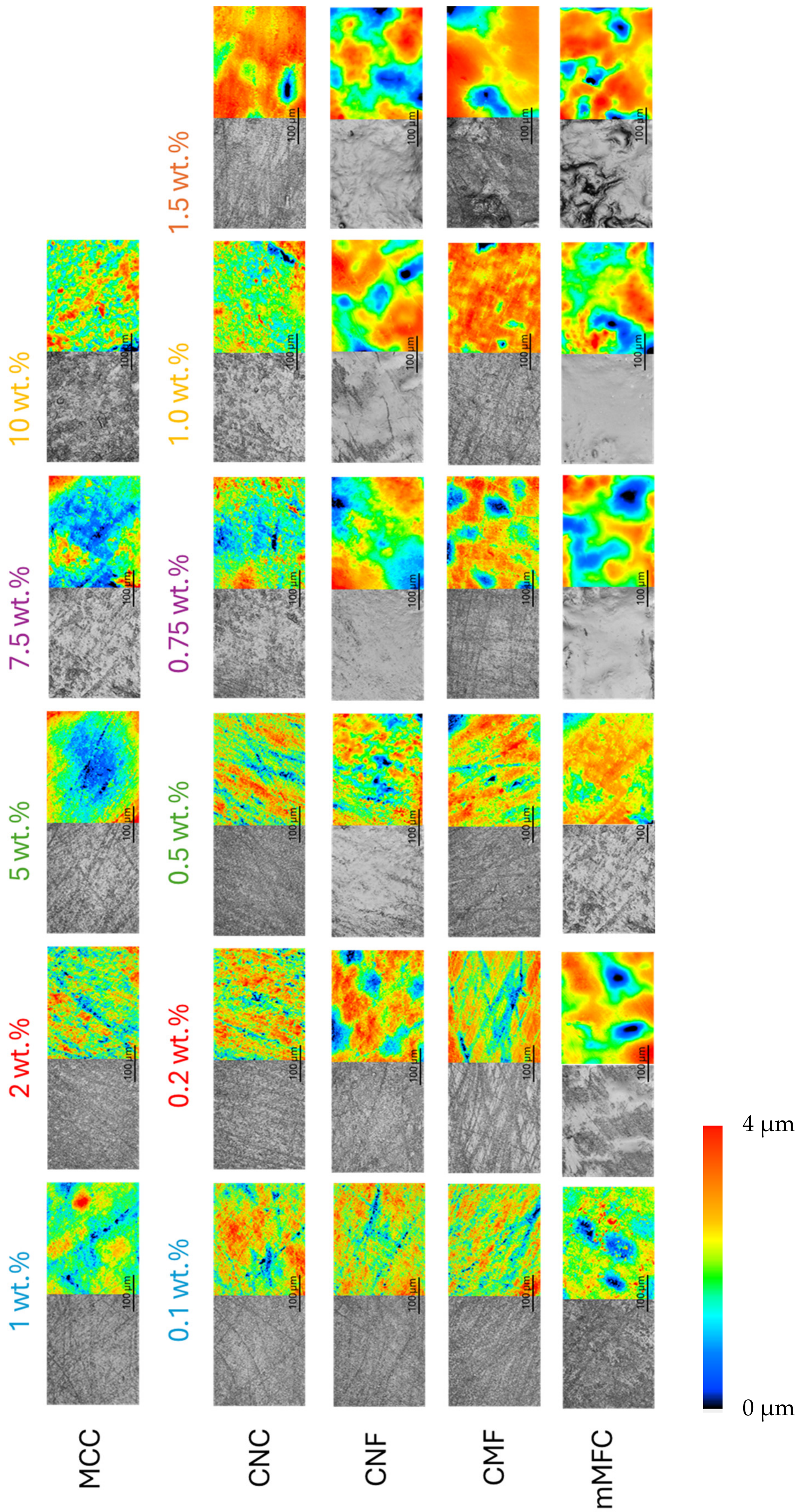

Figure S3. a. Surface morphologies (optical micrograph) and surface topography (3D scan) of worn epoxy coatings with different micro- and nanocellulose additives at various concentrations. Same color scale for z positions apply to all topographical images.

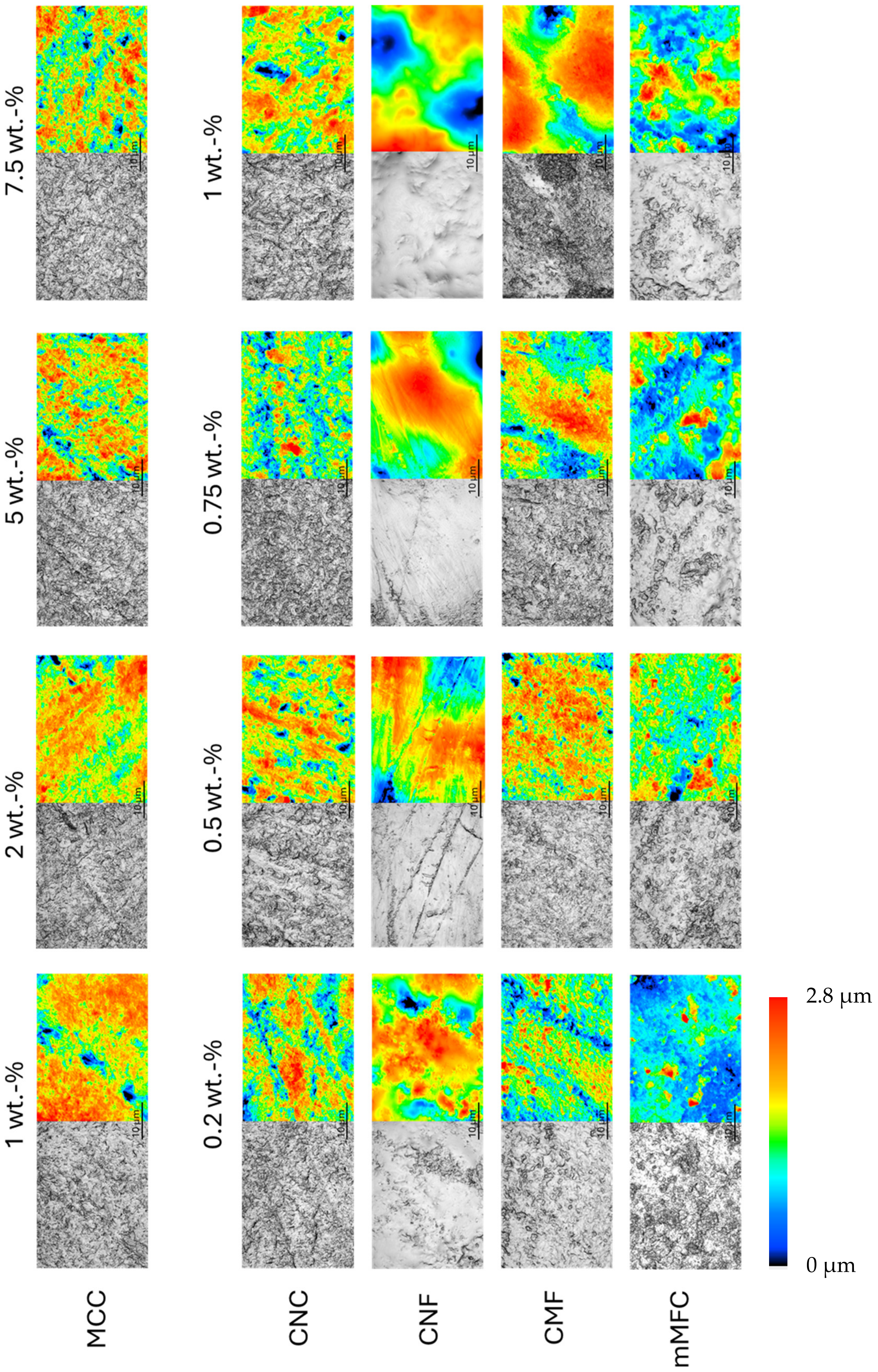

MCC
CNC
CNF
CMF
mMFC
1 wt.-%
2 wt.-%
5 wt.-%
7.5 wt.-%
0.2 wt.-%
0.5 wt.-%
0.75 wt.-%
1 wt.-%
2.8 μm
0 μm
10 μm

Figure S3. b. Detailed surface morphologies (optical micrograph) and surface topography (3D scan) of worn epoxy coatings with different micro- and nanocellulose additives at various concentrations. Same color scale for z positions apply to all topographical images.
